# Abstracts

**DOI:** 10.1155/S1463924685000037

**Published:** 1985


Journal of Automatic Chemistry, Volume 7, Number 1 (January–March 1985), pages 4–7

### Abstracts

### Page 8

### Automatic analyser/computer system for adaptive control of phosphate concentration during fermentation

M. Pécs *et al.*

An automatic on-line measuring and control system was developed for fermentation. An automatic analyser was used as the sensor for computer control. An optimal adaptive control algorithm was developed and tested for one component of a fermentation broth.

An autoanalyser was adapted to analyse the fermentation broth by continuous dialysis without pretreatment. Computer-controlled magnetic valves were used as a sampler unit. The photometer was connected to the computer through an analogue-digital converter.

Programs were developed in BASIC for handling the hardware, evaluating the input data and for adaptive control, and were run on a TRS 80 computer. The controller worked as an adaptive minimum variance controller.

The system was calibrated and tested with standard phosphate solutions and yeast suspension. Dynamic characteristics were investigated.

*Saccharomyces cerevisiae* (baker's yeast) fermentation was monitored by the system. Phosphate concentration was measured during a period in which the concentration changed from 20 to 300 mg/l. Cell growth and phosphate consumption occurred in parallel.

Phosphate concentration was controlled by the system during *S. cerevisiae* fermentations at preset levels: 40 and 50 mg /l. Both the measuring system and control software proved satisfactory (the measuring and control system working reliably for over 10 h of fermentation). There was no significant difference between measuring and controller variances in the experiment.

### Page 8

### Système analyseur automatisé pour réglage adaptive de concentration de phosphate en procès de fermentation

M. Pecs *et al.*

Les auteurs développaient un système on-line pour la mesure et le réglage automatisé des procès de fermentation. Un analyseur automatique fut appliqué en senseur pour le contrôle computerisé. Un algorithme d'adaptation optimalisé fut développé et éprouvé concernant un des components de liquide de fermentation.

Un analyseur automatique fut remanié pour analyser le liquide de fermentation par dialyse continuelle, sans traitement préalable. Pour prélever l'échantillon, des soupapes magnètiques furent appliquées, controllées par le computer. Le photomètre fut connecté au computer à travers d'un convertisseur digital-analogue.

Les programmes pour faire fonctionner le hardware, pour évaluer les signaux d'entrée et pour réaliser le contrôle adaptif, furent développés en langage BASIC, et ils furent traités par un computer TRS 80. Le contrôleur fonctionnait en adaptive minimum variance structure.

Le systéme fut calibré et essayé par solutions phosphate standard et par suspensions de levain. Les caractéristiques dynamiques furent aussi enregistrés.

La fermentation de *Saccharomyces cerevisiae* (levain de boulangerie) fut observée par cet appareil. Au cours des mesures, la concentration de phosphate changeait de 20 à 300 mg/l. La multiplication cellulaire et la consommation de phosphate se mouvait parallèlement.

La concentration de phosphate au cours de la fermentation de *Saccharomyces cerevisiae* fut contrôlée par ce systéme d'un niveau de signal 40 et 50 mg/l. Le systéme de mesure et aussi le software pour le contrôle remplirent les conditions. Le systéme de mesure et de contrôle fonctionnait sans défaut pendant la durée de 10 heures de fermentation. Entre les tolérances de systéme de mesure et celui de contrôle n'était pas une différence significante.

### Page 8

### Computergesteuertes Analisatorsystem für adaptive Steuerung von der Phosphatkonzentration in Fermentationsvorgängen

M. Pécs *et al.*

Zur Prüfung und Regelung von Fermentationsvorgängen hatten die Autoren ein automatisches on-line System entwickelt. Für Sensoranlage wurde ein automatischer Analysator angewendet. Es wurde auch ein optimalisierter adaptiver Regelungsalgorythmus entwickelt und erprobt auf einem der Komponenten der Fermentlösung. Es wurde ein automatischer Analysator umgebaut für vorbehandlungslose Untersuchungen der Fermentlösung mit kontinuierlicher Dialyse. Die Probeentnahme wurde durch computergesteuerte magnetische Ventile ausgeführt. Der Ausgang des Photometers wurde durch einen analog-digitalen Umsetzer zum Computer geleitet.

Die Programme zur Hardwarefunktion, zur Signalauswertung und zur adaptiven Regelung wurden auf BASIC geschrieben, und auf einem Computer TRS 80 aufgearbeitet. Die Regelung wurde als ein adaptiver Regler von minimaler Streuung ausgebildet. Das System wurde mit standardisierten Phosphatlösungen und mit Hefesuspensionen ausgeprüft bzw. kalibriert. Die dynamischen Eigenschaften wurden auch untersucht.

Mit dem System wurde die Fermentation von *Saccharomyces cerevisiae* (Backhefe) nachvollzogen. Die Phosphatkonzentration bewegte sich während der Messungen von 10 bis 300 mg/l. Die Zellenvermehrung und der Phosphatverbrauch änderten sich parallel.

Die Phosphatkonzentration während der Fermentation von *Saccharomyces cerevisiae* wurde mit dem System auf Grundsignalwert von 40 bis 50 mg/l geregelt. Sowohl die Messeinrichtung wie die Software für die Regelung erwiesen sich als einwandfrei. Beide Systeme arbeiteten während der Dauer von mehr als 10 Stunden des Fermentationsvorgangs mit hoher Zuverlässigkeit. Zwischen den Toleranzstreuungen von der Messeinheit und der Regelung ergab sich kein deutlicher Unterschied.

---

**Page 20**

**The derivation of reference values from a patient population in clinical chemistry**
J. B. Hemel *et al.*

An overview of some methods that derive reference intervals for clinical chemical variables from patient populations is given. The method proposed by Naus was studied and is discussed here in more detail. This method is based on the assumption that the variables are distributed either according to a Gaussian curve or to a Pearson's gamma distribution. To facilitate the choice between the normal and the gamma model, three additional plots are proposed in this study. Some restrictions based on experience with this technique are described and the results of application of the technique are presented with some critical remarks.

**Page 20**

**Discussion critique d'une méthode pour développement de limites de référence en chimie clinique à partir d'une population de malades**

J. B. Hemel *et al.*

Une revue de certaines méthodes qui permettent de développer des intervalles de référence pour des variables de chimie clinique de populations de malades est donnée. La méthode propagée par Naus a été étudiée et discutée en plus de détails. Cette méthode est basée sur l'hypothèse que les variables sont distribuées soit selon Gauss ou selon une distribution gamma de Pearson. Pour faciliter la choix entre le modèle normal ou le modèle gamma trois propositions additionnelles sont étudiées. Certaines restrictions basées sur l'expérience avec cette technique sont décrites et les résultats de l'application de la technique sont présentés avec quelques remarques critiques.

**Page 20**

**Kritische Diskussion einer Methode für die Herleitung von Referenz-Grenzwerten in der klinischen Chemie aus einer Patienten-Population**
J. B. Hemel *et al.*

Es wird eine Uebersicht gegeben über einige Methoden, die Referenz-Intervalle für klinisch-chemische Variablen von Patienten-Populationen herleiten. Die von Naus entwickelte Methode wurde untersucht und wird hier etwas detaillierter diskutiert. Diese Methode basiert auf der Annahme, dass die Variablen entweder Gaussisch oder nach der Pearson's Gammafunktion verteilt sind. Um die Wahl zwischen Normalverteilung und Verteilung nach dem Gamma-Modell zu erleichtern, werden in dieser Studie drei zusätzliche graphische Darstellungen vorgeschlagen. Auf einige Einschränkungen, die auf der Erfahrung mit dieser Technik basieren, wird hingewiesen und die Ergebnisse der Anwendung dieser Technik werden zusammen mit einigen kritischen Bemerkungen präsentiert.

---

**Page 31**

**A fully automatic apparatus for chemical reactions on the laboratory scale**
M. Legrand and P. Bolla

An automatic chemical set controlled by computer is described with some indications of the language designed for its use. This apparatus is useful for the optimization of reactions in the field of organic chemistry.

**Page 31**

**Un appareil complètement automatique pour réactions chimiques à l'échelle de laboratoire**

M. Legrand and P. Bolla

Un appareil chimique automatique contrôlé par ordinateur est décrit avec certaines indications concernant le langage développé pour son utilisation. Cet appareil est util pour l'optimisation de réactions dans le domaine de la chimie organique.

**Page 31**

**Ein vollautomatisches Gerät für chemische Reaktionen im Labormassstab**
M. Legrand and P. Bolla

Es wird eine automatisierte chemische Anlage mit Computersteuerung beschrieben mit Hinweisen auf die Sprache, die dafür erfunden wurde. Diese Anlage ist für die Optimierung·von Reaktionen im Gebiet der organischen Chemie nützlich.

---

**Page 38**

**The clinical biochemistry laboratory computer system as a simple calculator: a program in MUMPS**
T. G. Pellar *et al.*

The hand-held calculator is an efficient device for performing the simple arithmetical operations of addition, subtraction, multiplication and division. By contrast, the laboratory computer system is less efficient at this function. The authors have written a program in MUMPS that allows the terminals of the laboratory computer system to emulate a handheld calculator for these four common arithmetical operators. This program is now in daily use. It requires about 8·9 kbyte of core memory.

**Page 38**

**Le système d'ordinateurs pour laboratoires de biochimie clinique utilisé comme simple calculateur: un programme en MUMPS**
T. G. Pellar *et al.*

La petite calculatrice de poche est un instrument efficace pour les simples opérations arithmétiques qui sont l'addition, la soustraction, la multiplication et la division. Par contre, les ordinateurs de laboratoire sont nettement moins efficaces pour ces fonctions. Cette publication décrit un programme écrit en MUMPS qui permet aux terminaux du système d'ordinateurs de laboratoire de simuler une calculatrice de poche pour ces 4 fonctions arithmétiques simples. Ce programme est utilisé dans les laboratoires de l'hôpital universitaire de l'Ontario de l'Ouest. Il nécessite environ 8·9 kbyte de mémoire centrale.

**Page 38**

**Das klinisch-biochemische Laborcomputersystem als einfache Rechenmaschine: Ein Programm in MUMPS**
T. G. Pellar *et al.*

Der tragbare Taschenrechner ist ein effizientes Gerät um einfache arithmetische Operationen wie Addition, Subtraktion, Multiplikation und Division durchzuführen. Dazu im Gegensatz ist ein Laborcomputersystem in diesen Funktionen weniger effizient. Die vorliegende Arbeit berichtet über ein Programm, das in MUMPS geschrieben ist, das erlaubt, auf den Terminals des Laborcomputersystems die vier gewöhnlichen arithmetischen Operationen eines tragbaren Taschenrechners zu emulieren. Dieses Programm steht jetzt im täglichen Gebrauch im Laboratorium des University Hospital von Western Ontario. Es beansprucht ungefähr 8,9 kbyte Speicherplatz.

---

**Page 42**

**Use of N-(1-naphthyl)-ethylendiamine-dihydrochloride (NEDD) as secondary calibrator for conjugated bilirubin on the DuPont aca**
M. L. Gozzo *et al.*

N-(1-naphthyl)-ethylendiamine-dihydrochloride (NEDD) is suggested for calibrating the conjugated bilirubin method on DuPont's Automatic Clinical Analyser (aca). The authors' attempt to simplify the calibration procedure suggested by DuPont relies principally on the stability of NEDD solutions and on the assignment of 'apparent CBil' values to this material starting from a certified TBil calibrator. A split sample comparison with a reference method has been conducted after calibration of the aca using the NEDD, in order to confirm the accuracy of the proposed calibration procedure.

**Page 42**

**Utilisation du N-(1-naphthyl)-ethylendiamine-dihydrochloride (NEDD) comme calibrage secondaire pour le bilirubin conjugué mesuré avec le aca de DuPont**
M. L. Gozzo *et al.*

Le N-(1-naphthyl)-ethylendiamine-dihydrochloride (NEDD) est suggéré pour calibrer la méthode pour le bilirubin conjugué sur l'analyseur clinique automatique (aca) de DuPont. L'essai des auteurs de simplifier la procédure de calibrage suggérée par DuPont se base principalement sur la stabilité des solutions NEDD et sur la corrélation de valeurs CBil apparentes en partant d'un calibrateur certifié TBil pour ce matériel. Une comparaison d'échantillons séparés avec une méthode de référence a été conduite après calibrage du aca utilisant le NEDD afin de confirmer l'exactitude de la procédure de calibrage proposée.

**Page 42**

**Die Verwendung von N-(1-naphtyl)-ethylendiamin-dihydrochlorid (NEDD) als sekundäre Eichsubstanz für konjugiertes Bilirubin auf dem DuPont aca**
M. L. Gozzo *et al.*

N-(1-naphtyl)-ethylendiamindihydrochlorid (NEDD) wird vorgeschlagen für die Kalibrierung der konjugierten Bilirubin-Methode auf dem DuPont Automatic Clinical Analyzer (aca). Um das Eichprozedere, das von DuPont vorgeschlagen wird, zu vereinfachen, verlassen sich die Autoren hauptsächlich auf die Stabilität der NEDD Lösungen und auf die Zuordnung von scheinbaren CBil-Werten zu diesem Material, die auf einen TBil-Eichstandard zurückgeführt werden. Für die Bestätigung der Genauigkeit des vorgeschlagenen Eichprozederes wurde nach Kalibrierung des aca unter Verwendung von NEDD mit geteilten Proben und einer Referenzmethode ein Vergleich durchgeführt.

---

**Page 45**

**'Instant' cusums from a discrete analyser**
P. Henry and D. C. Ephraim

Cusums are the cumulative sums of successive differences from a target value. They are very sensitive indicators of error in an analytical system, but they have not been much used because they are tedious to calculate and evaluate manually.

A program is described which has been written for a microcomputer dedicated to printing out the results from a discrete analyser. The theory behind the program is given, together with a flow diagram and an example of the final print-out.

The advantage of the system is that it gives an up-to-date picture of the performance of the instrument, immediately after each control sample has been analysed. In addition, the results obtained for the last 10 similar control samples are reproduced. Thus both the person operating the analyser and the laboratory director have much more information readily available on which to take decisions.

**Page 45**

**Des cusums instantanés d'un analyseur discret**
P. Henry and D. C. Ephraim

Les cusums sont les sommes cumulatives des différences successives d'une valeur standard. Ils sont des indicateurs très sensibles de l'erreur d'un système analytique, mais ils n'ont pas été utilisés beaucoup car ils ils sont pénibles à calculer et à juger manuellement.

Un programme est décrit qui a été écrit pour microordinateurs dédiés pour imprimer les résultats d'un analyseur discret. La théorie utilisée pour ce programme est présentée ainsi qu'un diagramme de flux et un exemple du compte-rendu final.

L'avantage du système est qu'il donne une idée à jour de la performance de l'instrument immédiatement après l'analyse des échantillons de contrôle. En plus, les résultats obtenus pour les 10 derniers échantillons de contrôle similaires sont reproduits. De cette facon, la personne utilisant l'analyseur et le directeur de laboratoire ont beaucoup plus d'informations pour prendre leurs décisions.

**Page 45**

**Instant 'Cusums' eines diskreten Analysengerätes**
P. Henry and D. C. Ephraim

'Cusums' sind die kumulierten Summen aufeinanderfolgender Differenzen zu einem Zielwert. Sie sind ein sehr empfindlicher Indikator für Fehler in einem Analysensystem, aber wurden bisher nicht sehr häufig benützt, weil sie manuell mühsam zu berechnen und auszuwerten sind.

Es wird ein Programm vorgestellt, das für einen Mikrocomputer geschrieben wurde, der die Resultate eines diskreten Analysengerätes ausdruckt. Die Theorie, auf der das Programm aufbaut, wird auseinandergesetzt, und ein Flussdiagramm und ein Beispiel des abschliessenden Ausdrucks angeführt.

Der Vorteil des Systems liegt darin, dass laufend ein Bild der Leistungsfähigkeit des Gerätes gezeichnet wird, und zwar unmittelbar nach jeder Kontrollprobe. Zusätzlich werden die Resultate der letzten 10 ähnlichen Kontrollproben wiedergegeben. So haben die für das Gerät verantwortlichen Personen als auch der Direktor des Laboratoriums sehr viel mehr Informatioon zur Verfügung, um ihre Entscheide zu fällen.

### JAC IN THE USA

Margaret R. Stewart has been appointed Managing Editor: USA. Ms Stewart, who will commission articles in North America and promote the *Journal* there, holds a B.S. degree in Food Chemistry from the University of California at Berkeley and an M.S. degree in Food Chemistry from the Florida State University. She worked as a chemist in government, university, and hospital laboratories; taught chemistry at Fairleigh Dickinson University; and was a writer/editor for several USA government agencies. Most recently, Ms Stewart served as Staff Officer of several studies for the Food and Nutrition Board of the US National Research Council/National Academy of Sciences. She would welcome suggestions for research reports, reviews and evaluations and can be contacted at 14 Shawnee Trail, Blacksburg, Virginia 24060, USA.

Dr Kent K. Stewart has agreed to join *JAC*'s team of editorial advisers (he has been a 'corresponding editor' for some time). Dr Stewart received a B.A. in Chemistry from the University of California at Berkeley and a Ph.D. in Chemistry from the Florida State University. He joined the Rockefeller University in New York City in 1965, where he served as a Guest Investigator and later as a Research Associate and an Assistant Professor. In 1970 he joined the US Department of Agriculture (USDA) as a Research Chemist. In 1975 he was appointed the first Chief of the USDA Nutrient Composition Laboratory. In 1982, Dr Stewart was appointed to the position he currently holds, that of Professor of Food Science and Head of the Department at Virginia Polytechnic Institute and State University in Blacksburg, Virginia. Dr.Stewart's research and teaching interests are in the field of laboratory automation, flow-injection analysis (of which he is one of the inventors), liquid chromatography, and numerous aspects of the nutrient composition of foods.

Further details on the way the newly established North American Office will operate will be forthcoming in future issues of *Journal of Automatic Chemistry.*

### FORTHCOMING PAPERS

Later 1985 issues will include the following articles:

An inexpensive fast memory module for rapid acquisition of digital data
A comprehensive laboratory automation system
The changing role of computers in the laboratory
X-ray photon attenuation measurement as a technique for monitoring liquid composition
Assessment of the Eppendorf ERIS analyser
Calculation error following sample dilution: a proposal for processing such specimens using a MUMPS program
Evaluation of an automated haemolytic method for the determination of anti-Streptolysin O antibodies
The use of a microprocessor for flexible automation of an experimental procedure

